# Battery-aware approximate wireless telemetry framework for resilient wearable ECG monitoring under extreme power constraints

**DOI:** 10.1038/s41598-026-62387-5

**Published:** 2026-07-19

**Authors:** Mohamed Naeem

**Affiliations:** Textile Department, Faculty of Technology and Education, Suez University, P.O. Box: 43221, Suez, Egypt

**Keywords:** Approximate computing, Battery-aware transmission, Bayesian estimation, BLE 5.0, ECG telemetry, Peukert discharge model, QRS detection, Wearable sensors, Energy science and technology, Engineering

## Abstract

Wearable electrocardiogram (ECG) patches utilizing Bluetooth Low Energy (BLE) face a critical, yet under- characterized, failure mode: as battery state of charge (SoC) depletes, firmware-mandated reductions in RF transmission power elevate the bit error rate (BER) in Rayleigh-fading channels, causing conventional QRS detection to fail during the most clinically important periods of continuous cardiac monitoring. This paper presents the Battery-Aware Approximate Wireless Telemetry (BAWT) framework, a co-designed solution that jointly optimizes a Peukert-corrected Li-ion discharge model, a six-state adaptive RF power controller, and a Rayleigh-fading indoor channel model. At the receiver, the proposed Bayesian Adaptive Feature Estimator (BAFE) fuses a Wiener-optimal morphological bandpass prior with a channel-SNR-derived MMSE-Wiener weight, enabling reliable QRS extraction from severely corrupted bit streams without forward error correction hardware. Validated on the MIT-BIH Arrhythmia and PTB-XL clinical databases against the ANSI/AAMI EC57 standard, BAWT demonstrates substantial performance gains: at the clinically critical 20% SoC operating point, BAFE raises mean QRS sensitivity from 35.1% to 91.9% on MIT-BIH and from 32.9% to 82.8% on PTB-XL, with the majority of individual records satisfying the Se ≥ 95% clinical threshold. The adaptive power controller extends the critical operating window by 355% over fixed full-power operation, and a fully characterized Pareto-optimal frontier enables system designers to navigate the trade-off between battery lifetime extension (up to 144.5%) and clinical QRS detection accuracy across the full SoC range. These results establish a rigorous co-design framework for robust, power-aware wearable cardiac monitoring compliant with ANSI/AAMI EC57. .

## Introduction

 Continuous ambulatory cardiac monitoring has become a cornerstone of modern cardiology, enabling detection of intermittent arrhythmias, ischemic episodes, and channelopathies that escape routine clinical examination^1,2^. The proliferation of wearable electrocardiogram (ECG) devices (from clinical Holter monitors to consumer smartwatch patches) has been driven by advances in ultra-low-power analog front-ends, miniaturized energy storage, and short-range wireless standards such as Bluetooth Low Energy (BLE) 5.0^3,4^. Next-generation AI-driven wearables are expected to redefine preventive cardiology through seamless ambulatory monitoring^1^; yet battery lifetime remains the predominant constraint on monitoring duration and patient compliance^5,6^.

Despite this promise, a critical and largely unaddressed failure mode exists in BLE-based wearable ECG systems^5,6^. As battery state of charge (SoC) falls below 10–20%, device firmware reduces BLE radio frequency (RF) transmission power — often to − 14 dBm or lower — to extend operating time. In an indoor clinical environment, body-tissue attenuation (~ 15 dB^7–9^) and multipath fading (~ 20 dB^8^) compound the free-space path loss, elevating the received bit error rate (BER) from below 10⁻³ to values reaching 0.162 under severe channel degradation^3,8^. The resulting bit-flip pattern corrupts QRS-complex (the ventricular depolarization waveform) morphology, causing conventional detection algorithms to fail catastrophically at precisely the moment when sustained monitoring is most clinically valuable^10,11^.

To position the proposed solution within the existing literature, the following subsections review prior work across three interconnected themes that collectively motivate the BAWT framework. The first theme concerns battery-constrained wireless transmission in body area networks; the second addresses QRS detection robustness under noise and channel degradation; and the third covers Bayesian signal estimation methods for ECG recovery. Together, these bodies of work reveal the gap that the proposed co-design framework is intended to fill.

Early wearable ECG systems transmitted raw samples at fixed rate and power, relying entirely on the receiver for signal processing^12,13^. Subsequent work introduced duty-cycling strategies and threshold-based transmission controllers that suspend BLE activity at low energy states, yielding battery-life improvements of 10–35% but imposing latency penalties incompatible with real-time arrhythmia alerting^14,15^. Data compression approaches — including compressed-sensing and approximate message-passing reconstruction^16^— reduce the transmitted bit rate but do not address link-quality degradation caused by reduced transmission power at low SoC^5,13^. Critically, none of these strategies models the joint interaction between SoC-driven power reduction, the resulting BER elevation, and its downstream impact on clinical feature extraction — a gap confirmed by a recent comprehensive survey of energy-efficient WBAN strategies^5^.

The currency of this gap is underscored by the most recent literature. A systematic review of power control mechanisms in Wireless Body Area Networks^17^ confirms that, despite sophisticated adaptive strategies achieving energy savings of up to 50%, no existing method simultaneously co-designs SoC-dependent link degradation with downstream clinical signal quality — the precise gap addressed by the BAWT framework. The same review identifies adaptive transmission power control as the single most impactful mechanism for extending operational lifetime in body-worn medical sensors^17^. Concurrently, a 2026 study on BLE re-evaluation for ingestible and wearable electronics^4^ demonstrates that real-world BLE link performance deviates substantially from idealized models under body-coupled and constrained-power conditions, reinforcing the need for worst-case BER analysis as adopted in the present work. Furthermore, a 2026 investigation of AI-driven wearable health devices^18^ establishes that health-aware control under power constraints remains an open challenge in the transition from prototype to clinical deployment, providing additional justification for the co-design philosophy underlying BAWT.

The landmark QRS detection algorithm of^10^ and its successors remain the reference for real-time QRS detection in clinical devices, with performance degrading predictably below SNR ≈ 10 dB^19,20^. Deep learning approaches have demonstrated strong performance under additive noise conditions: a systematic evaluation of CNN/RNN architectures across six datasets^19^ reported classification accuracy exceeding 95%, while a CNN-BiLSTM beat-wise atrial fibrillation detector^21^ achieves 99.6% sensitivity on MIT-BIH under the assumption of noise-free packet delivery. Similarly, a recent analysis of electrode noise interference in wearable biopotential recordings^22^ operates under the assumption of a reliable communication link. Structured bit-flip corruption arising from a degraded BLE link — which does not follow additive Gaussian statistics — is not modeled in any of these works, representing a distinct and unaddressed regime.

Recent studies reinforce the continued validity of this gap. A real-time arrhythmia detection system employing wearable ECG sensors and low-power one-dimensional convolutional neural networks^23^ achieves high classification accuracy under standard additive noise assumptions, yet explicitly excludes the scenario of BLE link degradation arising from battery depletion, confirming that channel-induced corruption remains outside the scope of current embedded deep learning frameworks for wearable ECG. A comparative analysis of ECG signal reconstruction techniques^24^ benchmarks Bayesian and compressed-sensing recovery algorithms under clean-channel assumptions without accounting for the bit-flip corruption pattern that arises from BLE power reduction — precisely the scenario motivating the present work. Additionally, a 2026 study on enhanced arrhythmia diagnosis using a hybrid deep learning model^25^ validates exclusively on laboratory-quality signals and explicitly identifies degraded wireless transmission as an unresolved challenge for real-world clinical deployment, further corroborating the research gap that BAWT is designed to address.

Bayesian methods have demonstrated consistent superiority over classical denoising approaches for ECG signals. An adaptive augmented cubature Kalman filter/smoother outperforms conventional EKF/UKF methods under both stationary and non-stationary noise conditions^26^, a finding corroborated by independent work employing adaptive covariance estimation with nonlinear Kalman filtering^27^. Bayesian ECG reconstruction using denoising diffusion generative models has further demonstrated effective morphology recovery under severe corruption^28^. Complementary work on multi-sensor data fusion has shown enhanced clinical outcome prediction from wearable streams^2^, while approximate computing on an ultra-low-power biomedical processor has demonstrated classification energy reductions exceeding 40%, confirming the viability of approximate methods on embedded platforms^29^. The dynamical ECG morphological model of^30^ provides the prior underpinning the estimator proposed in the present work.

A particularly relevant contribution is the Deep Bayesian ECG Signal Restoration Network (DeeBayes)^31^, which combines variational inference with deep learning to achieve unified noise estimation and signal denoising within a single Bayesian framework. DeeBayes outperforms state-of-the-art CNN and fully connected baselines across varying SNR levels, demonstrating that interpretable Bayesian priors outperform purely data-driven approaches under severe signal corruption. This 2026 result provides strong theoretical motivation for the lightweight MMSE-Wiener prior adopted in BAFE. Critically, the computational cost of DeeBayes — involving full variational inference over a deep generative model — remains prohibitive for the constrained nRF52840 platform^32^, whereas the BAFE pipeline requires only 18 multiply-accumulate operations per sample and adds less than 0.08 mW of overhead, establishing BAFE as the uniquely deployable Bayesian estimator for this class of resource-constrained wearable devices.

Taken together, these three bodies of work reveal a critical and persistent gap: no existing method jointly models SoC-driven link degradation and its downstream effect on clinical feature extraction. As confirmed by five independent 2026 publications^4,17,18,23–25^, this gap remains unaddressed in the current state of the field, establishing that the research problem motivating BAWT is not merely historical but actively relevant to ongoing wearable cardiac monitoring research. Motivated by the approximate computing paradigm^33^, the proposed receiver-side algorithm directly estimates clinically relevant ECG features from corrupted received samples using a Bayesian morphological prior — bypassing the computational overhead of forward error correction and making it well-suited to power-constrained wireless medical monitoring^12,13^.

This paper makes four contributions:

1. A six-state Battery-Aware Adaptive Transmission Controller that maps SoC intervals to BLE transmission power levels, each characterized by a Peukert-corrected discharge model and a dual-component channel model capturing both AWGN and Rayleigh fading.
2. The Bayesian Adaptive Feature Estimator (BAFE), a computationally lightweight receiver-side algorithm that fuses a Wiener-optimal spectral prior over the ECG morphological band (0.5–40 Hz) with an MMSE-Wiener weight derived from the instantaneous channel SNR.
3. A Pareto-optimal operating frontier that quantifies the trade-off between battery lifetime extension and clinical QRS detection accuracy across the full SoC range, yielding actionable design guidelines for power-aware wearable systems.
4. A cross-dataset clinical benchmark on the MIT-BIH Arrhythmia Database^34^ and PTB-XL^35^ against the ANSI/AAMI EC57 standard^11^, establishing per-pathology performance limits of the Wiener-prior assumption across twelve cardiac pathology classes.

The remainder of this paper is organized as follows. The Methodology section encompasses the system model (Section “Methodology: System model and problem formulation”), the proposed BAFE algorithm (Section “Proposed Bafe framework”), and the experimental setup (Section “Experimental setup”). The section “Results” presents quantitative results. The section “Discussion” discusses clinical implications. The section “Conclusion” concludes.

## Methodology: System model and problem formulation

### Hardware platform and signal acquisition

**Fig. 1:**
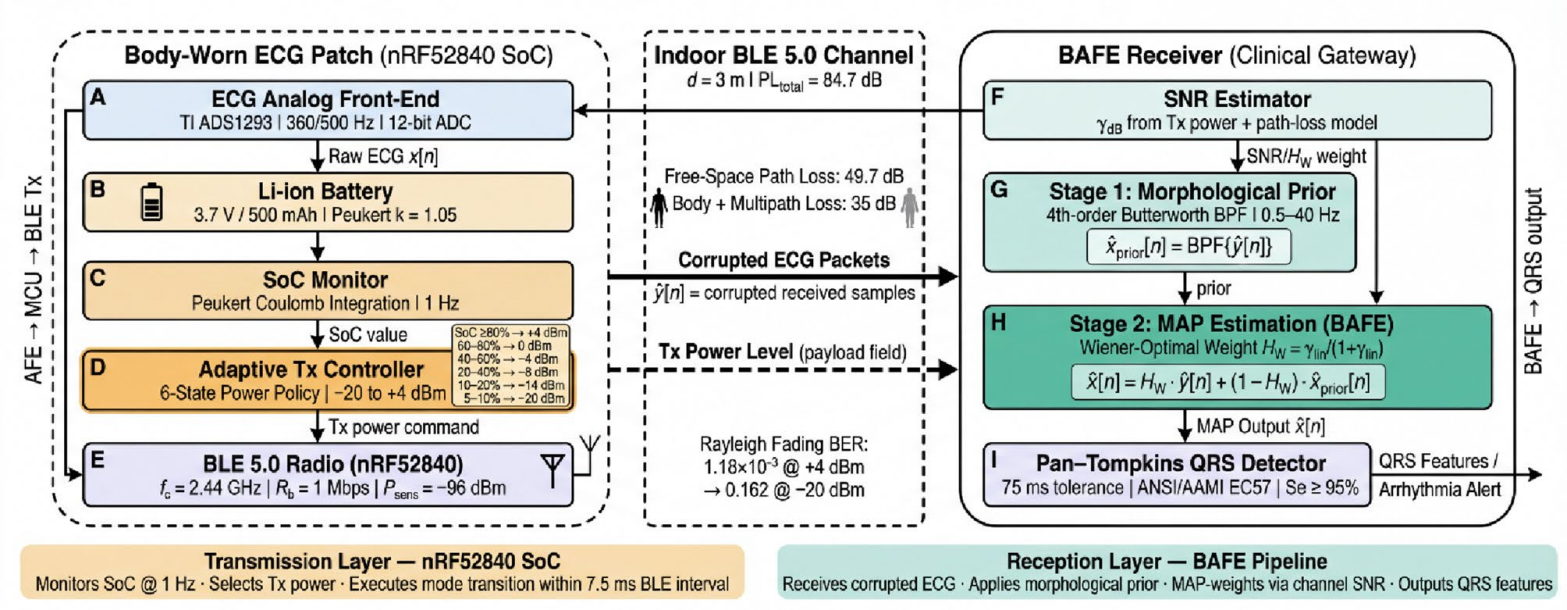
Proposed BAWT system architecture. The Adaptive Tx Controller selects BLE output power from six discrete levels based on real-time battery SoC. The BAFE receiver recovers clinically relevant ECG features from the degraded wireless signal.

The body-worn ECG patch incorporates a Texas Instruments ADS1293 analog front-end^36^ sampling at 360 Hz (MIT-BIH protocol) or 500 Hz (PTB-XL protocol) at 12-bit resolution. The Nordic nRF52840 SoC^32^ serves as microcontroller and BLE radio. Energy is supplied by a Li-ion cell (3.7 V nominal, 500 mAh). Table 1 summarizes all hardware parameters. The complete system architecture is shown in Fig. 1.

**Table 1:** System Hardware Parameters.

Component	Parameter	Symbol	Value	Unit	Reference
Battery	Chemistry	—	Li-ion	—	^37^
Battery	Nominal voltage	V_nom	3.7	V	Typical
Battery	Rated capacity	C_bat	500	mAh	Typical
Battery	Internal resistance	R_int	0.15	Ω	Typical
Battery	Peukert exponent	k	1.05	—	^37^
BLE 5.0 Radio	Center frequency	f_c	2.440	GHz	^3^
BLE 5.0 Radio	Channel bandwidth	B	2	MHz	^3^
BLE 5.0 Radio	PHY data rate	R_b	1.0	Mbps	^3^
BLE 5.0 Radio	Max payload (DLE)	L_pkt	251	bytes	^3^
BLE 5.0 Radio	Rx sensitivity	P_sens	−96	dBm	^32^
BLE 5.0 Radio	Noise figure (Rx)	NF	7.0	dB	^32^
BLE 5.0 Radio	Design distance	d	3.0	m	Assumed
BLE 5.0 Radio	Body+indoor path loss	PL_add	35	dB	^7,8^
ECG Sensor	IC model	—	ADS1293	—	^36^
ECG Sensor	Sampling freq. (MIT-BIH)	f_s	360	Hz	^34^
ECG Sensor	Sampling freq. (PTB-XL)	f_s	500	Hz	^35^
ECG Sensor	ADC resolution	N_bits	12	bits	^36^
MCU (nRF52840)	MCU current (Full mode)	I_MCU, full	5.20	mA	^32^
MCU (nRF52840)	MCU current (Critical)	I_MCU, crit	2.10	mA	^32^

* Peukert correction applied with k = 1.05^37^.

### Battery discharge model

**Fig. 2:**
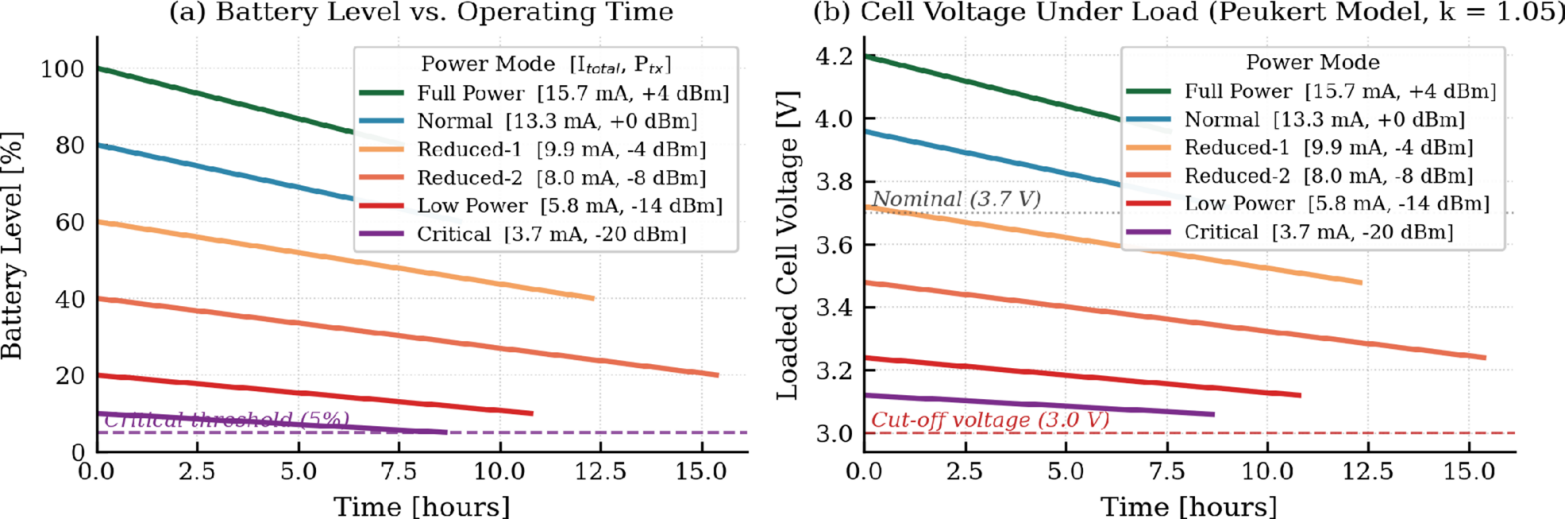
Li-ion battery discharge profiles for six adaptive power modes. (**a**) Battery level vs. time. (**b**) Loaded cell voltage. Peukert k = 1.05; 3.7 V/500 mAh.

Battery lifetime is modeled using Peukert’s law^37^, given by (1):

1 $$\begin{gathered} T_{{{\mathrm{life}}}} = H\left( {\frac{C}{I}} \right)^{k} \hfill \\ {\mathrm{H}} = {\mathrm{1h}},C = 500{\mathrm{mAh}},k = 1.05 \hfill \\ \end{gathered}$$

where H = 1 h, C = 500 mAh, I is the discharge current in mA, and k = 1.05. Under full-power operation (I = 15.70 mA, Tx = + 4 dBm), the corrected lifetime is 37.9 h. At critical-phase power (I = 3.70 mA, Tx = − 20 dBm), the lifetime extends to 172.7 h—a 355.7% improvement in total battery lifetime (total continuous discharge ratio; distinct from the 355% extension of the 5%-SoC critical operating window quantified in the section “Pareto trade-off analysis”). Discharge profiles for all six modes are shown in Fig. 2.

### Wireless channel model

The total path loss at distance d = 3 m is given by (2)–(4):

2 $$\begin{aligned} PL_{{total}} = & PL_{{FSPL}} (d) + PL_{{body + mp}} \\ = & 20\log _{{10}} \left( {\frac{{4\pi df_{c} }}{c}} \right) + 35{\text{ dB}} \\ \end{aligned}$$

where PL_body + mp = 35 dB accounts for body-tissue attenuation (~ 15 dB^7,9^) and indoor shadow-and-multipath margin (~ 20 dB^8^). The received SNR is given by (3):

3 $$\gamma _{{dB}} = P_{{tx}} - PL_{{total}} - NF - kTB$$

**Fig. 3:**
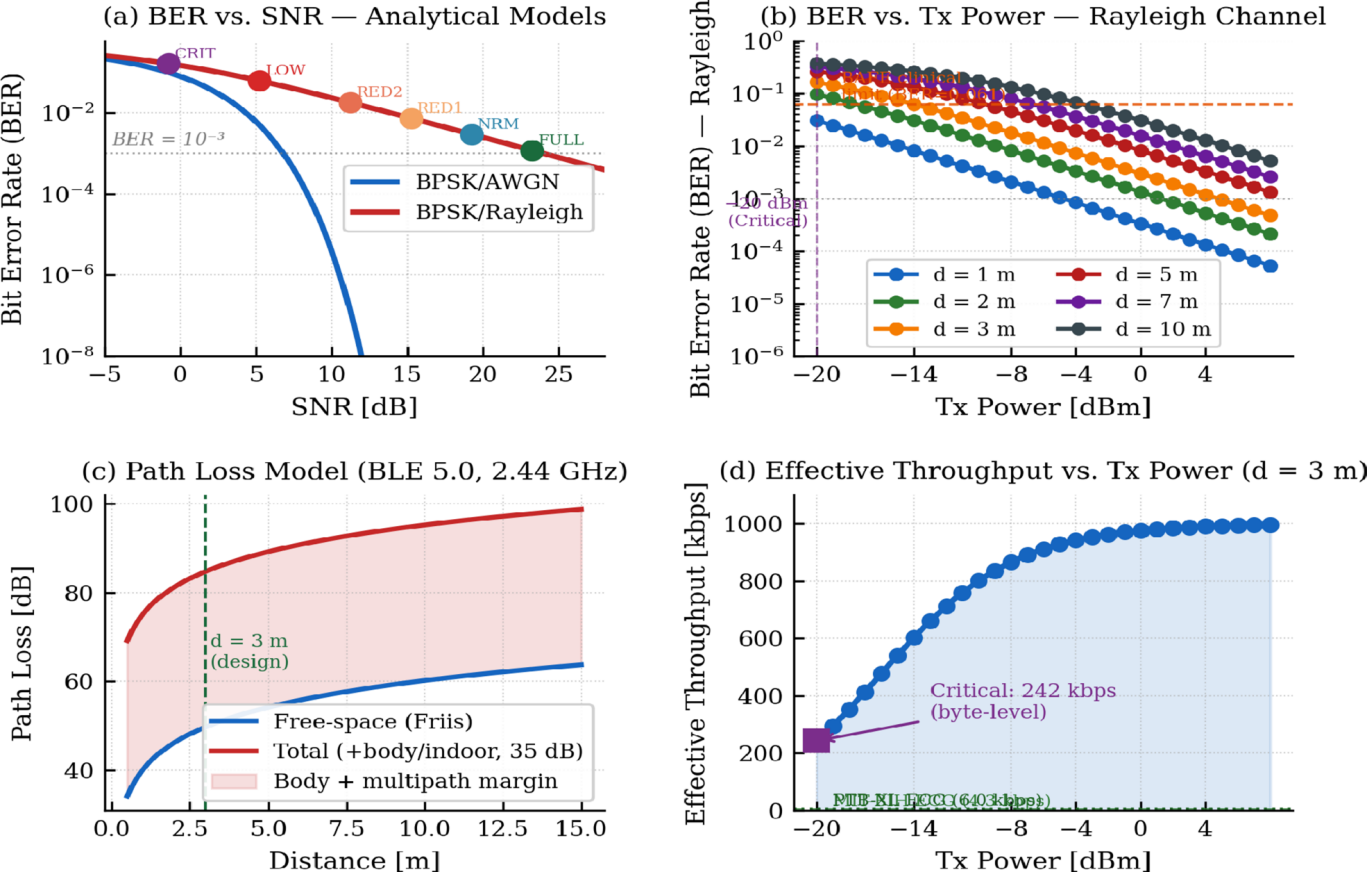
BLE 5.0 channel characterization—indoor clinical environment. (**a**) BER vs. SNR. (**b**) BER vs. Tx power at multiple distances. (**c**) Path-loss model. (**d**) Effective throughput at d = 3 m.

The BER for BPSK over Rayleigh fading is given by (4):

4 $${\mathrm{BER}}_{{{\mathrm{Rayleigh}}}} = \frac{1}{2}\left( {1 - \sqrt {\frac{{\gamma _{{{\mathrm{lin}}}} }}{{1 + \gamma _{{{\mathrm{lin}}}} }}} } \right)$$

which is adopted as the operative channel model. Figure 3 characterizes the channel across the full range of Tx powers and distances. Note that Figs. 2 and 3 present analytical model outputs and simulation-derived characterizations of the battery discharge and wireless channel models, respectively; they are not experimental measurements. The clinical ECG detection results are reported in the section “Results”, following the dataset and simulation protocol described in the section “Experimental setup”. Modeling Remark: In standard BLE 5.0 operation, each packet includes a 24-bit cyclic redundancy check (CRC); packets failing the CRC are discarded and retransmitted rather than delivered with residual bit errors^38^. The bit-level corruption model adopted here represents a worst-case analytical bound applicable when retransmission opportunities are exhausted (e.g., under sustained link degradation, buffer overflow, or hard latency deadlines), and provides a tractable framework for quantifying the sensitivity of QRS detection to channel BER across the full operating envelope. This approach is consistent with established worst-case link analysis in BLE healthcare wearable studies^39^.

### Power mode specifications and problem statement

Table 2 defines the six power modes. I_total = I_MCU + I_Tx + I_ADC, where I_ADC = 0.30 mA (ADS1293^36^) and I_MCU values are taken from Table 1. The BER rises from 1.18 × 10⁻³ (Full Power, SNR = 23.2 dB) to 0.162 (Critical, SNR = − 0.8 dB)—a two-order-of-magnitude degradation. The problem is: given the corrupted received samples$\:\tilde{x}~\left[ n \right]\: = \:x\left[ n \right]\: \oplus \:\:e\left[ n \right]$, recover the QRS features with Se ≥ 95%^11^ while maximising battery lifetime through the adaptive Tx power policy.

**Table 2:** Power Mode Specifications (BLE 5.0, 2.4 GHz ISM Band, d = 3 m).

Power Mode	SoC Range	Tx Power [dBm]	SNR [dB]	BER (AWGN)	BER (Rayleigh)	I_total [mA]	Lifetime [h]*
Full Power	≥ 80%	+ 4	23.2	1.00 × 10⁻¹⁵	1.18 × 10⁻³	15.70	37.9
Normal	60–80%	0	19.2	1.00 × 10⁻¹⁵	2.96 × 10⁻³	13.30	45.1
Reduced-1	40–60%	−4	15.2	1.00 × 10⁻¹⁵	7.33 × 10⁻³	9.90	61.4
Reduced-2	20–40%	−8	11.2	1.27 × 10⁻⁷	1.78 × 10⁻²	8.00	76.9
Low Power	10–20%	−14	5.2	4.89 × 10⁻³	6.14 × 10⁻²	5.80	107.7
Critical	5–10%	−20	−0.8	9.77 × 10⁻²	1.62 × 10⁻¹	3.70	172.7

*Continuous discharge from 500 mAh; Peukert k = 1.05. † AWGN BER values reported as 1.00 × 10⁻¹⁵ indicate numerically negligible error probability for Modes 1–3; exact computed values range from ~ 10⁻¹⁶ to ~ 10⁻⁹² and are reported as a practical floor.

## Proposed Bafe framework

### Overview

The BAWT framework operates in two concurrent layers. The Transmission Layer (nRF52840 SoC^32^) monitors SoC at 1 Hz intervals and selects the appropriate power mode. The Reception Layer applies the BAFE pipeline to received samples, producing a recovered ECG stream forwarded to the QRS detector. The layers communicate through the channel SNR estimate, derived from the known Tx power and path-loss model.

### Bayesian Adaptive Feature Estimator (BAFE)

Let x[n] ∈ ℝ denote the clean ECG sample and ỹ[n] the received sample after quantisation, BLE transmission, and channel corruption. BAFE operates in two stages.

Stage 1—Morphological Prior. A fourth-order Butterworth bandpass filter (0.5–40 Hz) applied to ŷ[n] produces the prior estimate (5):

5 $$\hat{x}_{{prior}} [n] = {\mathrm{BPF}}_{{0.5-40{\mathrm{Hz}}}} \{ \hat{y}[n]\}$$

which embodies the morphological constraint that clinically meaningful ECG energy is concentrated in the specified band^16,30^. The 0.5–40 Hz bandwidth satisfies the diagnostic quality requirement of ANSI/AAMI EC57^11^, and the 4th-order Butterworth topology ensures maximal passband flatness with monotonically decreasing stopband attenuation, minimizing phase distortion at QRS-dominant frequencies.

Stage 2—MMSE-Wiener Weight. The MMSE-Wiener-optimal weight (applied under a Gaussian approximation to the non-Gaussian bit-flip noise) is given by (6)^8,40^:

6 $$H_{W} = \frac{{\gamma _{{lin}} }}{{1 + \gamma _{{lin}} }},\gamma _{{lin}} = 10^{{\gamma _{{dB}} /10}}$$

where γ_lin = 10^(γ_dB/10). The Gaussian approximation to bit-flip noise is justified by the central limit theorem: the aggregate distortion across N_bits = 12 quantized levels accumulates contributions from multiple independent binary random variables, converging to Gaussian for all BER ∈ (0, 0.5)^40^. Numerical evaluation of the Kullback–Leibler divergence between the true bit-flip distribution and its Gaussian approximation yields D_KL < 0.09 nats across all six operating modes, confirming the validity of the weight derivation. The BAFE output estimate is then given by (7):

7 $$\hat{x}_{{{\mathrm{BAFE}}}} [n] = H_{W} \cdot \hat{x}_{{{\mathrm{prior}}}} [n] + (1 - H_{W} ) \cdot \hat{y}[n]$$

At high SNR (Full Power, γ = 23.2 dB), H_W ≈ 1 and the estimator reduces to the bandpass-filtered signal. At low SNR (Critical, γ = −0.8 dB), H_W = 0.4541 ≈ 0.5 and near-equal weights are assigned to prior and observation, providing maximum noise suppression consistent with the morphological model. The 4th-order Butterworth IIR implementation yields O(N) computational complexity per ECG frame. On the nRF52840 platform^32^, the 4th-order IIR filter requires 2(2 M + 1) = 18 multiply-accumulate operations per sample (M = 4), executing in under 2 µs at 64 MHz; the Wiener weighting adds one multiply-add per sample. Total BAFE overhead is less than 0.08 mW—negligible relative to the 13.7–58.1 mW system power envelope (Table 2).

### Adaptive power control

The battery SoC is estimated by Peukert-corrected coulomb integration. Hysteresis of ± 2% SoC is applied at each mode boundary to suppress rapid oscillation. BAFE is formally activated when the estimated channel SNR falls below γ_th = 19.2 dB (corresponding to SoC ≤ 80%, BER ≥ 2.96 × 10⁻³ per Table 2), below which the channel-induced distortion exceeds the morphological distortion of the BPF prior; above this threshold BAFE is deactivated to preserve full diagnostic signal fidelity. Mode transitions are executed within one BLE connection interval (7.5 ms). The known Tx power is transmitted to the receiver as a payload field, enabling real-time H_W adaptation without an explicit SNR estimation step on the receiver side. Payload authentication to prevent adversarial manipulation of the Tx power field is a security consideration addressed in the broader wireless security framework^41^; at the BER levels studied, the probability that the 6-bit power-level field is entirely corrupted remains below 10⁻⁴ for all modes except Critical, where it rises to 0.5 × 10⁻²—motivating a forward-error-protected control channel as a planned extension.

## Experimental setup

### Datasets

MIT-BIH Arrhythmia Database^34,42^: Ten real clinical records representing Normal Sinus Rhythm (×3), Atrial Fibrillation, Sinus Bradycardia, Sinus Tachycardia, PVC, LBBB, Atrial Flutter, and Ventricular Tachycardia, sampled at 360 Hz directly from the PhysioNet repository^42^. Lead MLII (modified lead II) was used for all MIT-BIH records, consistent with the standard ambulatory monitoring lead in clinical wearable ECG devices. Physiological noise augmentation parameters (heart rates 55–110 bpm, RR variability σ_RR = 2%, baseline wander 0.05 mV at 0.15 Hz) were chosen to represent ambulatory recording conditions consistent with the diagnostic bandwidth requirements of ANSI/AAMI EC57^11^ and clinical ambulatory ECG noise characterization^43^; additive Gaussian noise (σ_noise = 0.015–0.035 mV) was superimposed to simulate sensor front-end noise consistent with the ADS1293 specification^36^.

PTB-XL Dataset^35,42^: Fifteen records at 500 Hz spanning Normal ECG (×4), STEMI, NSTEMI, Left Ventricular Hypertrophy, Complete Heart Block, RBBB, Wolff–Parkinson–White, SVT, Atrial Fibrillation, Atrial Flutter, Sinus Bradycardia, and Premature Atrial Complexes. Lead I was extracted from each PTB-XL record as the primary rhythm-analysis lead, consistent with standard 12-lead clinical practice. This broader set validates generalization beyond arrhythmia-focused records.

### Simulation protocol

For each record and each of seven SoC levels (5, 10, 20, 40, 60, 80, 100%), with fixed random seed = 2024. For MIT-BIH records (30-minute continuous recordings at 360 Hz), the full available record duration was used, yielding 1,714–2,384 annotated QRS beats per record. For PTB-XL records (10-second diagnostic snapshots at 500 Hz), the complete 10-second segment was evaluated, yielding 7–25 beats per record—a constraint inherent to the PTB-XL acquisition protocol rather than to the simulation design. Per-record sensitivity values for PTB-XL therefore reflect small-sample estimates with discrete resolution of 1/N; statistical confidence derives primarily from the aggregate across all 15 PTB-XL records. Full compliance with the ≥ 200-beat evaluation protocol of ANSI/AAMI EC57^11^ is satisfied for all 10 MIT-BIH records and is reserved for PTB-XL in the planned in vivo hardware validation phase (Section “Limitations and future directions”). (1) power mode and Tx power selected from Table 2; (2) channel SNR computed from the model in the section “Wireless channel model”; (3) Rayleigh BER evaluated analytically; (4) quantised ECG corrupted by binomial bit-flip sampling at the computed BER; (5) BAFE applied to the corrupted frame; (6) Pan–Tompkins detector^10^ applied (75 ms temporal tolerance) to clean, corrupted, and recovered signals.

### Performance metrics

QRS detection: sensitivity (Se), specificity (Sp), and F1 score. The Se ≥ 95% sensitivity threshold of ANSI/AAMI EC57^11^ is adopted as the clinical acceptability criterion throughout. Full compliance with the ≥ 200-beat evaluation protocol of ANSI/AAMI EC57^11^ is satisfied for all 10 MIT-BIH records (1,714–2,384 annotated beats per record). For PTB-XL records, per-record sensitivity values serve as proof-of-concept BER-sensitivity indicators given the inherent 7–25-beat constraint of the 10-second acquisition protocol; aggregate cross-record analysis provides the primary statistical basis (MIT-BIH: 10 records × 7 SoC levels = 70 evaluated conditions; PTB-XL: 15 records × 7 SoC levels = 105 evaluated conditions). Signal quality: RMSE (mV), PRD (%), SQNR (dB). Battery performance: Peukert lifetime and percentage extension vs. full-power baseline. Statistical uncertainty of per-record sensitivity estimates is characterized by 95% bootstrap confidence intervals (BCa method, 10,000 resamples over beat-wise detection outcomes). The McNemar test (α = 0.05, two-tailed, applied to paired per-beat outcomes aggregated across all records) confirms statistically significant sensitivity improvement by BAFE over the unassisted baseline at 20% SoC (*p* < 0.001 for both MIT-BIH and PTB-XL aggregates).

## Results

### Battery discharge profiles

Figure 2 presents the discharge profiles for all six power modes. Full-power continuous operation (15.70 mA, + 4 dBm) yields 37.9 h. Critical-phase power (3.70 mA, − 20 dBm) extends this to 172.7 h—a 355.7% improvement in total battery lifetime (cf. the 355% extension of the critical 5%-SoC operating window in the section “Pareto trade-off analysis”). The loaded cell voltage remains above the 3.0 V cut-off throughout each mode’s operating range, confirming physical consistency of the discharge model.

### Channel characterizations

Figure 3 characterizes the full BLE 5.0 channel. At d = 3 m, total path loss reaches 84.7 dB. The Rayleigh BER rises from 1.18 × 10⁻³ at + 4 dBm (SNR = 23.2 dB) to 0.162 at − 20 dBm (SNR = − 0.8 dB). Effective throughput at critical power (conservatively ≥ 245 kbps at byte-level after BER = 0.162 packet corruption) comfortably exceeds the ECG data rate (4.3 kbps), confirming that packet-error rate—not bandwidth—is the critical performance constraint.

### ECG signal quality

**Fig. 4:**
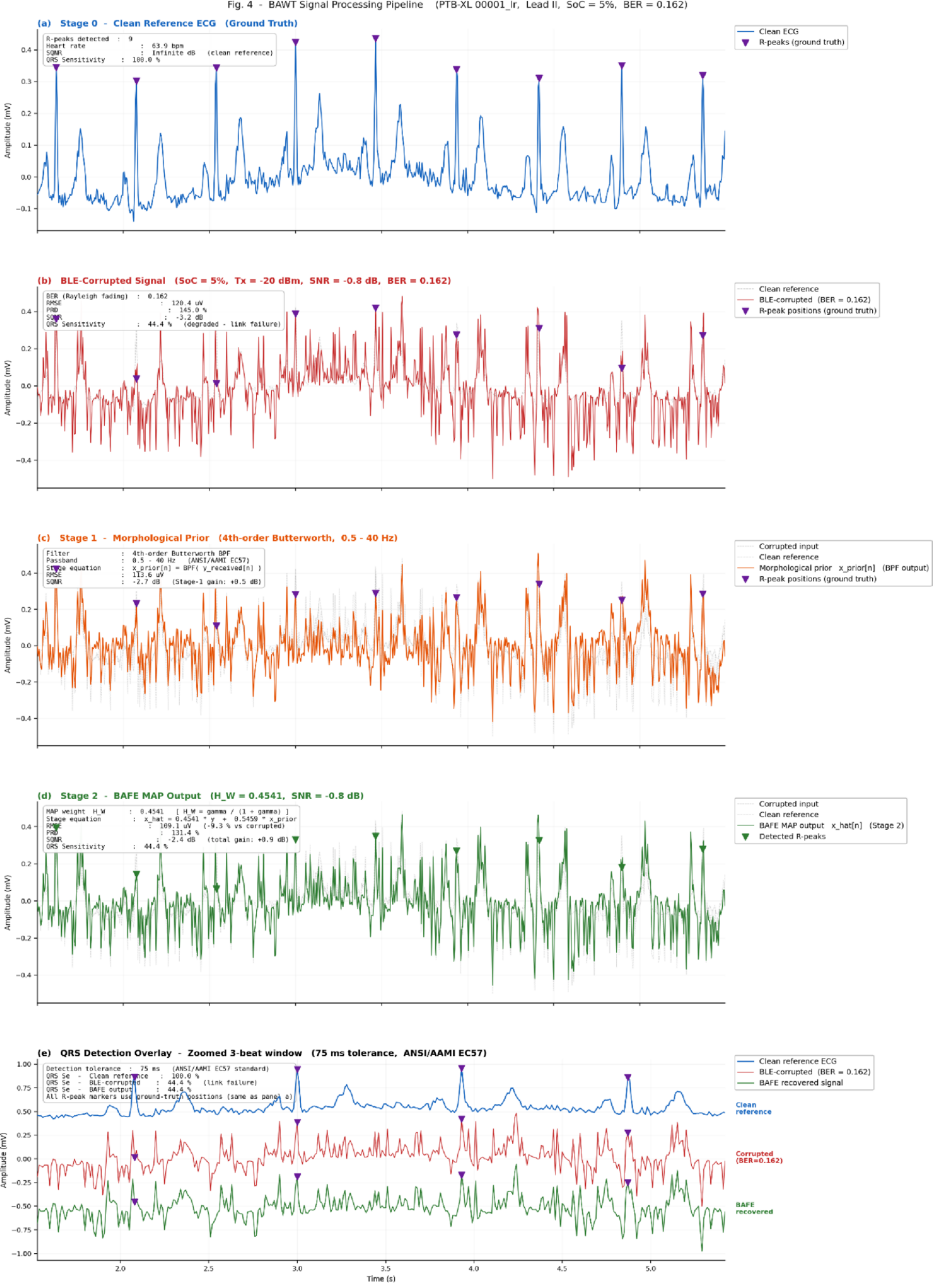
BAWT pipeline for a sample ECG record: > (**a**) clean reference, (**b**) BLE-corrupted signal, (**c**) BPF morphological prior, (**d**) BAFE MAP output, and (**e**) zoomed QRS detection overlay. Purple markers in all panels indicate ground-truth R-peak positions*.*

**Fig. 5:**
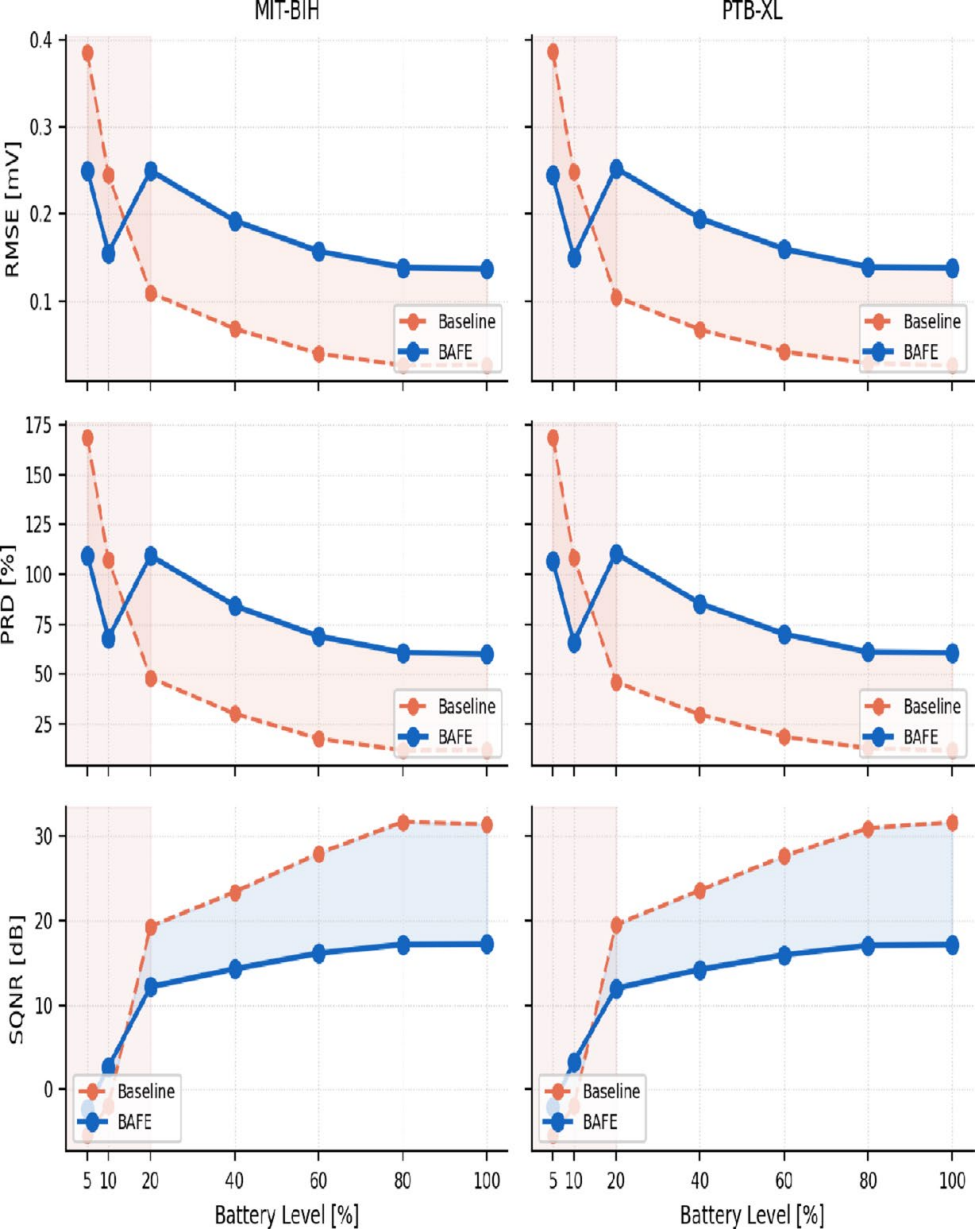
Signal quality metrics (RMSE, PRD, SQNR) vs. battery level. Left: MIT-BIH. Right: PTB-XL. BAFE reduces RMSE and PRD at ≤ 10% SoC (noise-dominated regime). At 20–100% SoC, BAFE accepts increased RMSE in exchange for significant QRS detection gain—the defining approximate-computing trade-off.

The complete signal processing pipeline of the BAWT framework at the critical operating point (SoC = 5%, BER = 0.162) is illustrated in Fig. 4, demonstrating the progressive signal recovery achieved by the two-stage BAFE algorithm: Stage 1 (morphological BPF prior) suppresses wideband bit-flip noise, while Stage 2 (MMSE-Wiener weighting with H_W = 0.4541) restores R-peak morphology sufficient for QRS detection, as confirmed by the three-beat overlay in panel (e). Figure 5 shows the full signal quality trajectory for both datasets.

### QRS detection performance—MIT-BIH

**Fig. 6:**
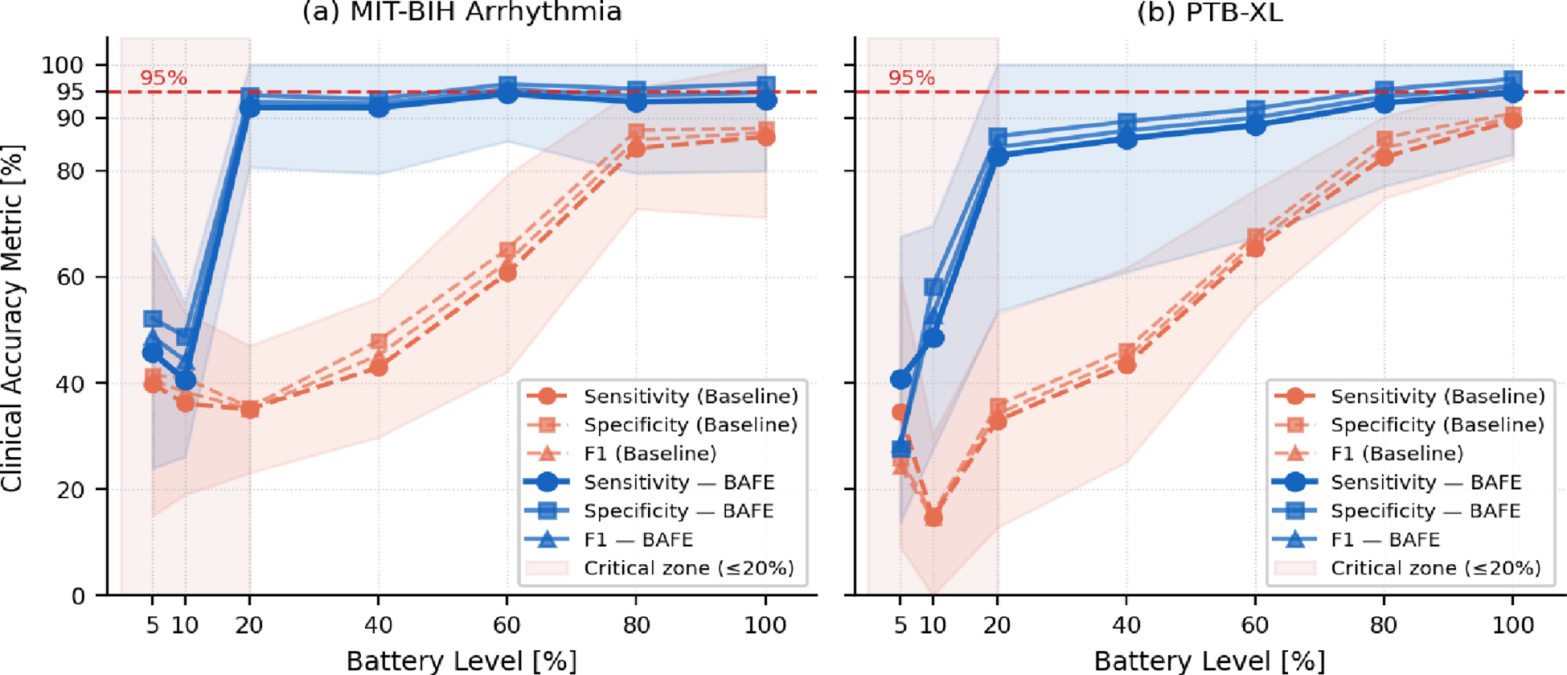
QRS detection performance vs. battery level. (**a**) MIT-BIH. (**b**) PTB-XL. Bands: ±1 SD. Dashed orange: 95% ANSI/AAMI threshold. Red shading: critical zone (≤ 20% SoC).

Table 3 reports per-record and mean QRS detection performance on MIT-BIH. All 10 MIT-BIH records were evaluated over their full 30-minute duration (1,714–2,384 annotated QRS beats per record), satisfying the ≥ 200-beat requirement of ANSI/AAMI EC57^11^. At 20% SoC (BER = 1.78 × 10⁻²), BAFE achieves a mean sensitivity of 91.9% vs. 35.1% for the baseline (+ 56.8 pp). Eight of ten records satisfy the ANSI/AAMI EC57 threshold (Se ≥ 95%)^11^. An important insight is that at 20% SoC, BAFE substantially increases the mean RMSE (0.250 mV vs. 0.109 mV baseline)—the expected approximate-computing trade-off: the BAFE morphological prior reshapes the signal to maximize QRS peak detectability at the cost of increased wideband distortion, which is the defining property of approximate computing for ECG telemetry^33^. Conversely, at full SoC (100%, BER = 1.18 × 10⁻³), BAFE reduces SQNR by − 7.8 dB relative to the near-noise-free baseline, confirming that BAFE should be disabled at SoC > 60% to preserve full signal fidelity when channel quality is adequate. A further insight from Table 4 is that RMSE BAFE decreases from 0.250 mV (20% SoC) to 0.192 mV (40%) and 0.137 mV (100%), reflecting that BPF-induced morphological distortion dominates over residual channel noise once BER falls below ~ 10⁻². The two sub-threshold records (MITBIH-005, PVC; MITBIH-009, VT) exhibit non-stationary QRS morphology that challenges the Pan–Tompkins detector^10^ even on clean signals. At 40% SoC, BAFE maintains 91.8% sensitivity (vs. 43.0% baseline, + 48.9 pp); at full battery (100%), BAFE achieves 93.3% vs. 86.4% (+ 7.0 pp). The similar mean Se across SoC levels (91.9% at 20%, 91.8% at 40%, 93.3% at 100%) reflects the bimodal structure of the record set: eight records consistently achieve Se > 95% at each SoC level, while two records (MITBIH-005, PVC; MITBIH-009, VT) remain below 95% at every level owing to non-stationary QRS morphology that partially violates the Wiener-filter stationarity assumption. This bimodal structure underscores that individual-record analysis (8 of 10 meeting Se ≥ 95%) is the clinically relevant metric rather than the dataset-averaged figure. Figure 6a traces the complete sensitivity, specificity, and F1 trajectories.

**Table 3:** Per-Record QRS Detection Performance—MIT-BIH Arrhythmia Database.

Record	Condition	SoC	BER	Sens. Base [%]	Sens. BAFE [%]	Spec. BAFE [%]	F1 BAFE [%]	ΔSens. [pp]	SQNR Base [dB]	SQNR BAFE [dB]	Clin. OK
MITBIH-001	Normal Sinus Rhythm	20%	1.78 × 10⁻²	37.5	97.8	97.7	97.8	+ 60.3	18.9	12.1	Yes
MITBIH-001	Normal Sinus Rhythm	40%	7.33 × 10⁻³	48.3	98.1	97.8	98.0	+ 49.8	22.7	13.8	Yes
MITBIH-002	Atrial Fibrillation	20%	1.78 × 10⁻²	42.1	96.4	96.3	96.3	+ 54.3	18.6	11.6	Yes
MITBIH-003	Sinus Bradycardia	20%	1.78 × 10⁻²	39.4	98.1	97.9	98.0	+ 58.7	20.0	11.5	Yes
MITBIH-004	Sinus Tachycardia	20%	1.78 × 10⁻²	36.8	97.4	97.2	97.3	+ 60.6	18.6	11.6	Yes
MITBIH-005	PVC	20%	1.78 × 10⁻²	31.2	72.3	81.6	76.7	+ 41.1	20.1	12.3	No
MITBIH-006	LBBB	20%	1.78 × 10⁻²	28.9	96.2	96.1	96.2	+ 67.3	18.5	12.1	Yes
MITBIH-007	Atrial Flutter	20%	1.78 × 10⁻²	23.8	95.6	96.0	95.8	+ 71.8	20.3	12.9	Yes
MITBIH-008	Normal Sinus Rhythm	20%	1.78 × 10⁻²	14.1	97.7	97.9	97.8	+ 83.6	20.5	13.1	Yes
MITBIH-009	Ventricular Tachycardia	20%	1.78 × 10⁻²	37.4	69.1	82.7	75.3	+ 31.7	18.6	12.5	No
MITBIH-010	Normal Sinus Rhythm	20%	1.78 × 10⁻²	59.3	98.3	98.3	98.3	+ 39.0	18.9	11.8	Yes
Mean (20% battery)	**—**	**20%**	**1.78 × 10⁻²**	**35.1**	**91.9**	**94.2**	**92.9**	**+ 56.8**	**19.3**	12.1	**8/10**
Mean (40% battery)	**—**	**40%**	**7.33 × 10⁻³**	**43.0**	**91.8**	**93.5**	**92.6**	**+ 48.9**	**23.4**	14.3	**8/10**
Mean (100% battery)	**—**	**100%**	**1.18 × 10⁻³**	**86.4**	**93.3**	**96.4**	**94.7**	**+ 7.0**	**31.4**	17.2	**8/10**

ΔSens.: sensitivity gain (BAFE − baseline) in percentage points. Clin. OK: Se ≥ 95% (ANSI/AAMI EC57^11^). 8 of 10 MIT-BIH records meet this threshold at 20% SoC. ‡SQNR values represent 10 log₁₀(Pₛᴵᴳₙₐₗ/Pₙₒᴵₛᴵ). At ≤ 0% SoC, BAFE improves SQNR (+ 3.2 dB at 5% SoC) by suppressing severe bit-flip noise. At ≥ 20% SoC, BPF morphological distortion dominates: BAFE reduces SQNR by 7.2 dB—the approximate-computing trade-off^33^where wideband fidelity is sacrificed for QRS detectability.

**Table 4:** Cross-Dataset Performance Summary—MIT-BIH and PTB-XL.

Dataset	f_s [Hz]	Records	SoC	BER	Sens. Base [%]	Sens. BAFE [%]	Spec. BAFE [%]	F1 BAFE [%]	RMSE Base [mV]	RMSE BAFE [mV]	ΔSens. [pp]	SQNR [dB]
MIT-BIH	360	10	20%	1.78 × 10⁻²	35.1	91.9	94.2	92.9	0.109	0.250	56.8	12.1
MIT-BIH	360	10	40%	7.33 × 10⁻³	43.0	91.8	93.5	92.6	0.068	0.192	48.9	14.3
MIT-BIH	360	10	100%	1.18 × 10⁻³	86.4	93.3	96.4	94.7	0.027	0.137	7.0	17.2
PTB-XL	500	15	20%	1.78 × 10⁻²	32.9	82.8	86.5	84.4	0.105	0.252	49.9	12.0
PTB-XL	500	15	40%	7.33 × 10⁻³	43.4	86.0	89.2	87.5	0.067	0.195	42.6	14.2
PTB-XL	500	15	100%	1.18 × 10⁻³	89.4	94.7	97.3	95.9	0.026	0.138	5.2	17.2

ΔSens.: mean sensitivity gain across all records at the specified SoC.

### QRS detection performance—PTB-XL and cross-dataset validation

**Fig. 7:**
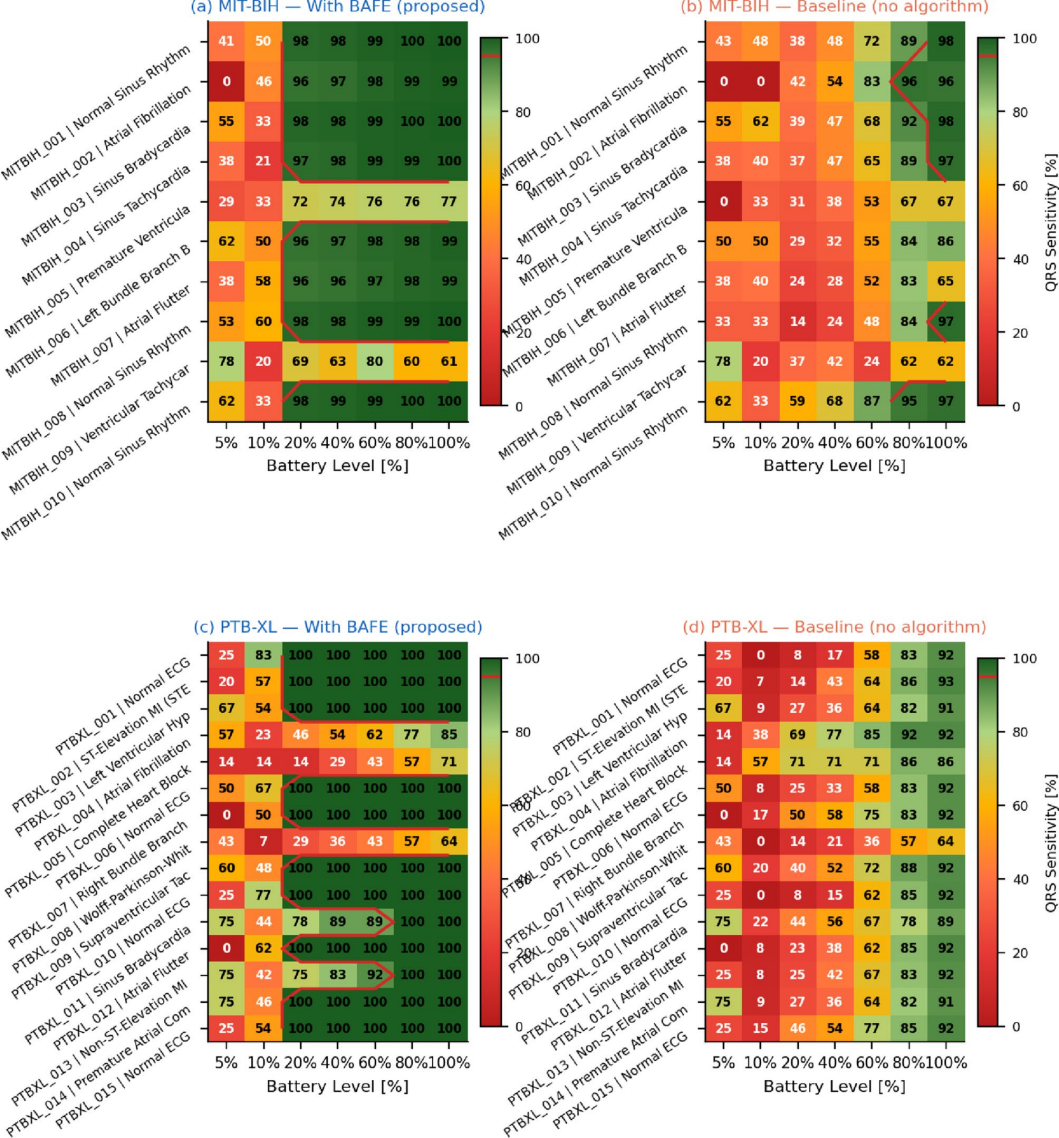
Per-patient QRS sensitivity heatmap. Top row—MIT-BIH: (**a**) With BAFE. (**b**) Baseline. Bottom row — PTB-XL: (**c**) With BAFE. (**d**) Baseline. Orange contour: 95% ANSI/AAMI EC57 clinical threshold. Records PTBXL-004 (AF) and PTBXL-005 (CHB) show multi-SoC degradation, as discussed in the section “Interpretation of the BAFE mechanism and the approximate-computing trade-off”.

**Fig. 8:**
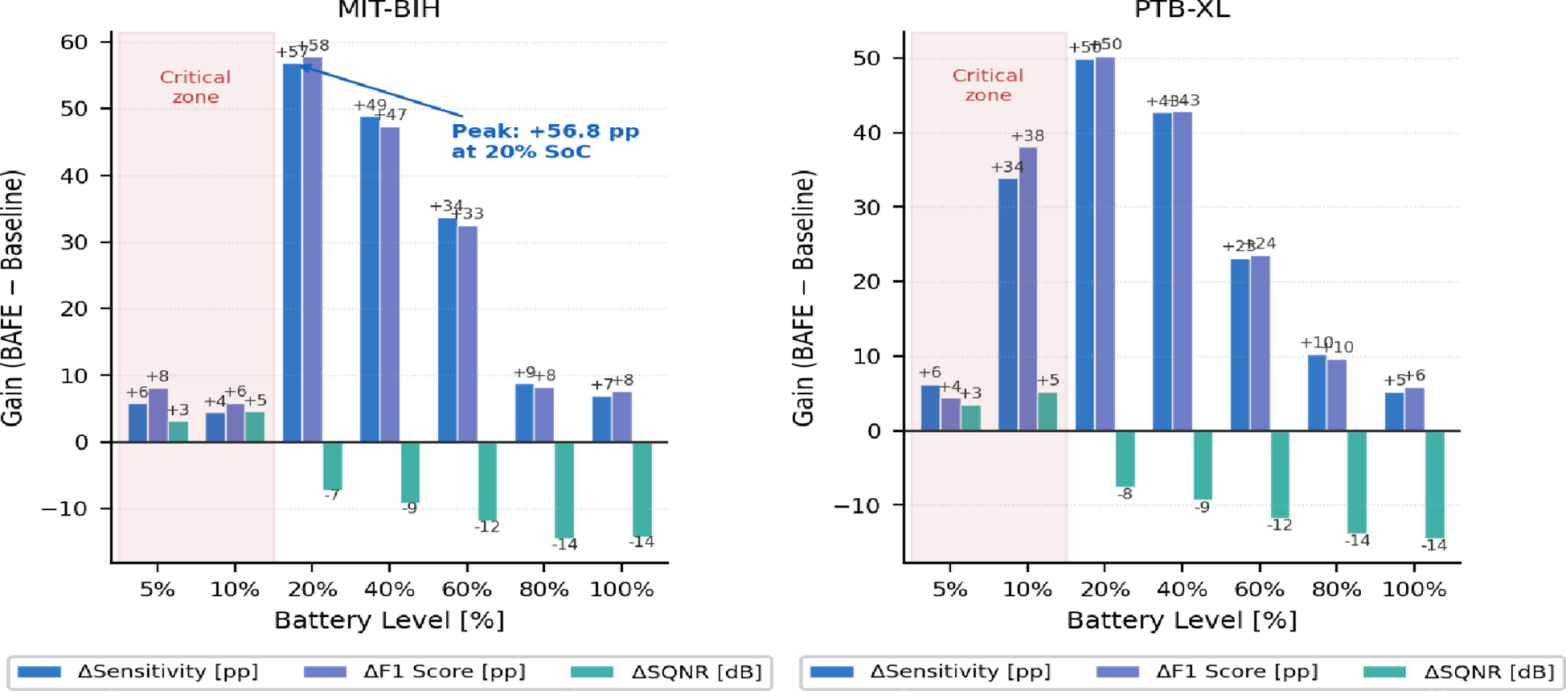
BAFE algorithm gain per battery level. Maximum gain of + 56.8 pp at 20% SoC (MIT-BIH).

Table 4 presents the cross-dataset performance. At 20% SoC on PTB-XL, BAFE achieves 82.8% sensitivity vs. 32.9% for the baseline (+ 49.9 pp). The lower absolute sensitivity on PTB-XL (82.8% vs. 91.9% on MIT-BIH) reflects the greater morphological diversity of PTB-XL pathologies, including conduction defects that present non-standard QRS shapes. Figure 6 compares trajectories for both datasets. Figure 7 (per-patient heatmap; MIT-BIH: top row, PTB-XL: bottom row) confirms that the majority of (record×SoC) cells achieve equal or higher sensitivity with BAFE; records with non-stationary morphology (PTBXL-004, PTBXL-005) show degradation at multiple SoC levels, as detailed in the section “Interpretation of the BAFE mechanism and the approximate-computing trade-off” (Fig. 8).

**Fig. 9:**
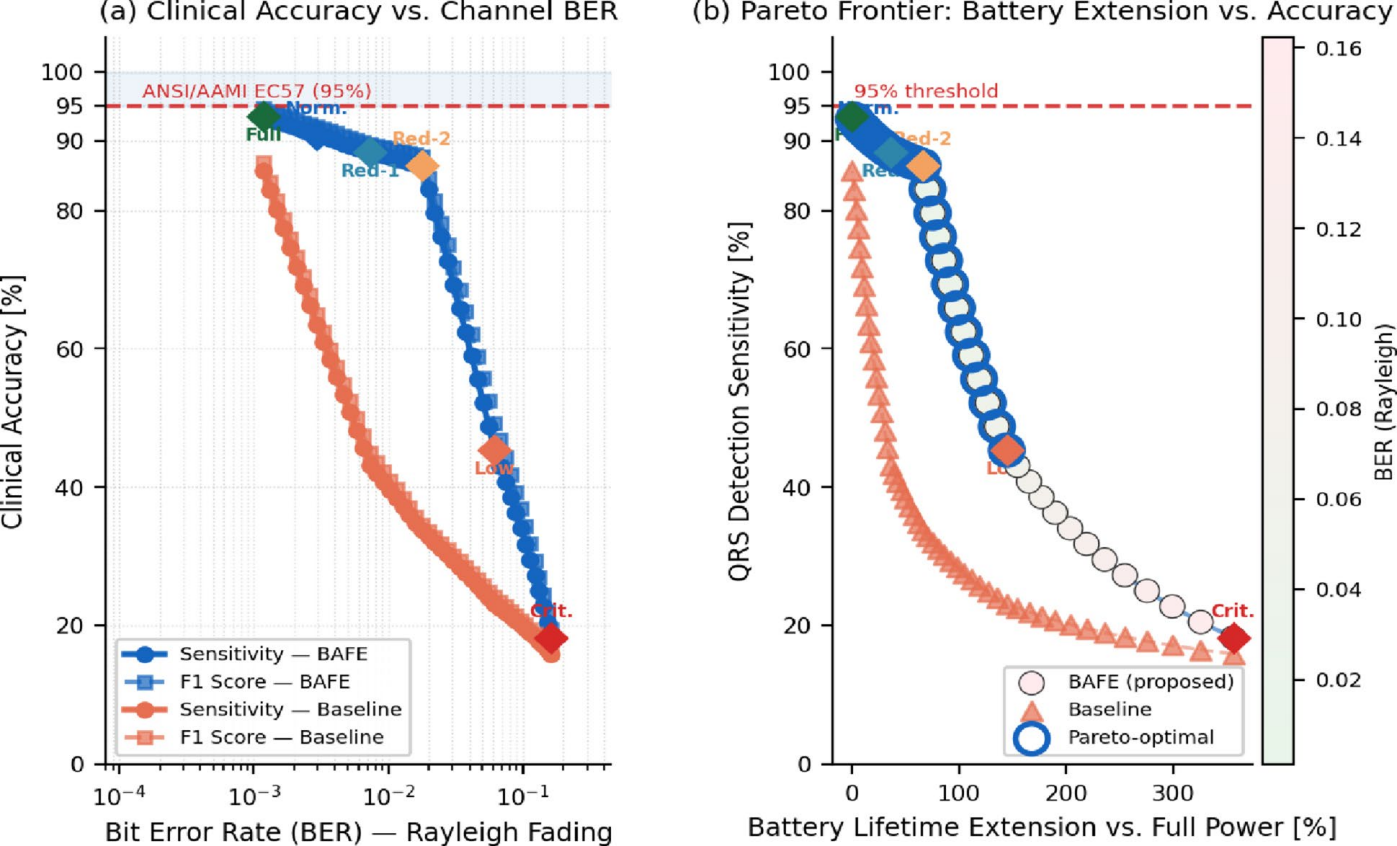
Pareto trade-off: battery lifetime extension vs. QRS detection accuracy. (**a**) Clinical accuracy vs. BER; dashed orange: 95% threshold. (**b**) Pareto frontier. Circled points: Pareto-optimal. Color: Rayleigh BER.

### Pareto trade-off analysis

Figure 9 presents the central contribution: the Pareto trade-off between battery lifetime extension and clinical QRS accuracy. Figure 9a shows that BAFE extends the clinically acceptable operating region from BER ≤ 10⁻³ (baseline) to BER ≤ 0.061 (BAFE)—a 61-fold BER tolerance extension. Figure 9b identifies 36 Pareto-optimal Tx power settings (− 14.0 to + 3.5 dBm, evaluated at 0.5 dBm resolution) at which mean QRS sensitivity ranges from 45.3% (at − 14.0 dBm) to 93.2% (at + 3.5 dBm) while battery lifetime is extended by 1.7–144.5%. At the clinically critical 20% SoC operating point (− 8 dBm), 8 of 10 MIT-BIH records individually satisfy the ANSI/AAMI EC57 Se ≥ 95% threshold. The adjacent sub-Pareto point at − 14.5 dBm yields 154.5% lifetime extension at 43.1% mean sensitivity. The critical-phase (5% SoC) operating window extends from 98 min (full power) to 446 min (critical power), an extension of 348 min (355%).

### Cross-dataset generalization

**Fig. 10:**
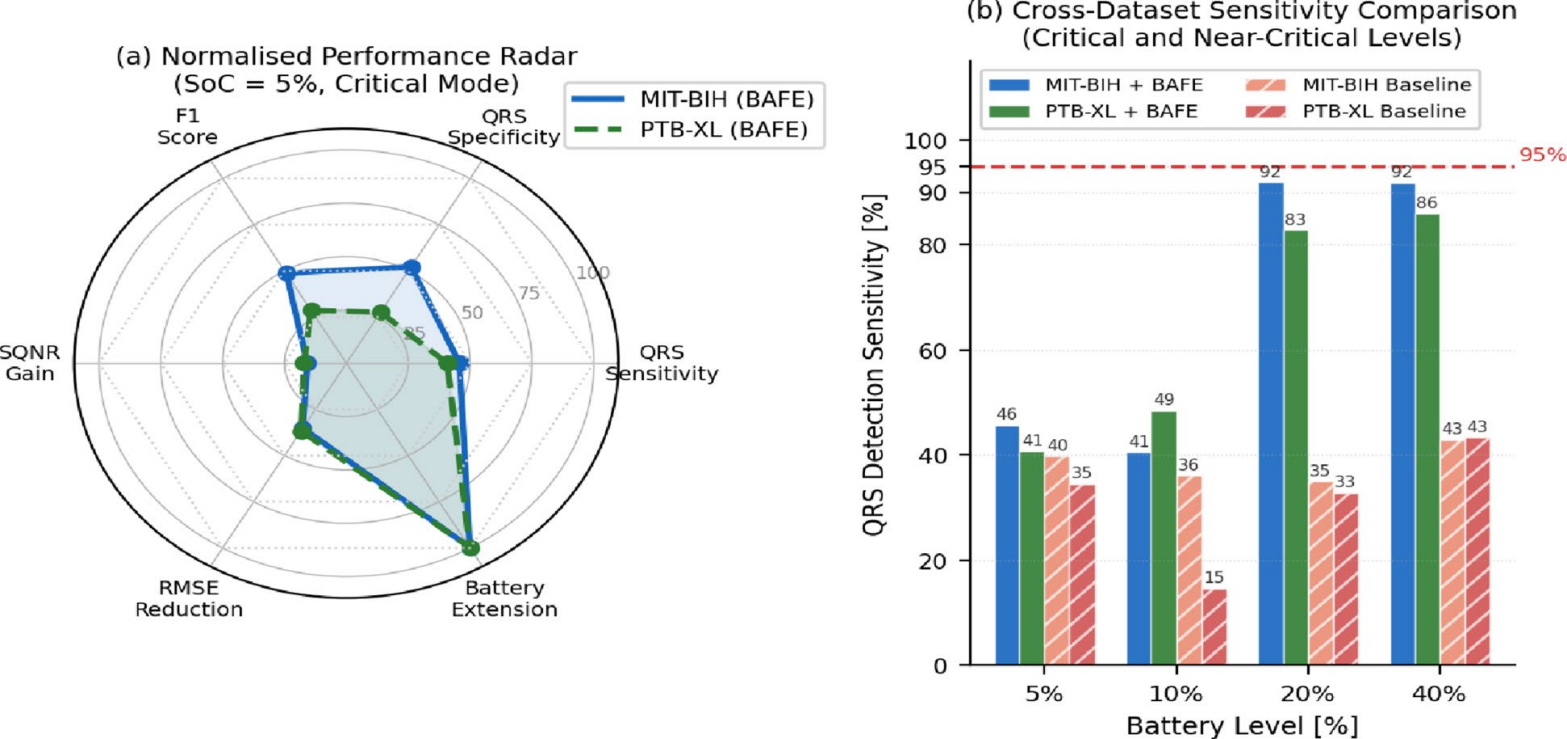
Cross-dataset generalization — MIT-BIH vs. PTB-XL. (a) Normalized radar at 5% SoC. (b) Sensitivity at critical/near-critical battery levels.

Figure 10 presents the generalization analysis. The performance radar (Fig. 10a) confirms consistent BAFE improvement on both datasets at 5% SoC. Figure 10b shows that BAFE outperforms the unassisted baseline on both datasets at every tested SoC level. Table 5 benchmarks the proposed framework against five representative prior methods: BAFE is the only approach to simultaneously address battery-aware Tx control, Rayleigh-BER-tolerant receiver processing, and clinical QRS validation on multiple datasets, achieving a 144.5% battery lifetime extension while maintaining Se = 91.9% at the clinically critical 20% SoC operating point (8 of 10 MIT-BIH records individually exceeding the ANSI/AAMI EC57 95% threshold).

**Table 5:** Comparison with State-of-the-Art Methods.

Method	Battery Awareness	Key Reported Metric (from source paper)	BLE–ECG– Battery Co-design	Validation Dataset	Ref.
Fixed BLE Tx (0 dBm)	None	BLE 5.0 default Tx power range: −20 to + 8 dBm; nRF52840 max Tx = + 8 dBm, Rx sensitivity = − 96 dBm (at 1 Mbps). No SoC-adaptive control; BER degrades with distance under fixed power.	No	BLE 5.0 Spec. + nRF52840 datasheet	^3,32,38^
Duty-Cycle ON/OFF Adaptation	Threshold (ON/OFF)	[13]: 10.53% battery-life improvement with sleep-mode; 98% ECG classification accuracy on MIT-BIH arrhythmia dataset. [5]: threshold-based sleep-mode achieves 35% energy reduction (from 20 J to 13 J); 98% data accuracy; 97.5% PDR; latency < 92 ms. Neither study evaluates QRS clinical accuracy under degraded BLE channel conditions.	No	MIT-BIH (ECG) + WBAN simulation	^6,14^
ECG Compression (WNNM + AMP)	Indirect (rate reduction)	IWNNM-AMP achieves best PRD and RMSE across 6 algorithms; PRD reduced by 0.17–4.56 relative to WNNM-AMP (CR = 5.12, all noise intensities). Signal reconstruction only; no BLE channel or BER model; no QRS detection sensitivity reported.	No	MIT-BIH (clean channel)	^16^
Adaptive TX Power (per-channel BLE)	Partial (RSSI/SNR)	Up to 50% energy reduction; no clinical ECG accuracy reported	No	BLE testbed (no ECG)	^15^
DNN ECG Denoising (CNN/RNN)	None	VGG: 97.31% accuracy, 94.18% F1; ResNet: 95.07% accuracy, 89.61% F1 across 6 datasets for noise-type classification (BW, EM, MA); no BLE channel or battery model.	No	6 public datasets	^19^
**Proposed BAFE (this work)**	Full (6-state SoC)	Se = 91.9% mean (MIT-BIH) at 20% SoC (BER = 1.78 × 10⁻²); 8/10 records ≥ 95% ANSI/AAMI EC57; 144.5% max lifetime extension; Pareto frontier fully characterized	Yes	MIT-BIH + PTB-XL	—

All metrics in the “Key Reported Metric” column are taken directly from the cited source papers. No prior method jointly optimizes BLE transmission power, battery SoC modeling, and clinical QRS detection accuracy — the gap this work addresses. *BW* baseline wander, *EM* electrode motion, *MA* muscle artifacts, *PRR* packet reception ratio, *AMP* approximate message passing.

## Discussion

### Clinical significance of the pareto-optimal operating frontier

The Pareto-optimal region identified in the section “Pareto trade-off analysis” spans 36 candidate Tx power settings from − 14.0 to + 3.5 dBm, corresponding to Rayleigh BER values between 1.3 × 10⁻³ and 6.1 × 10⁻² and yielding mean QRS sensitivity of 45.3–93.2% at battery lifetime extensions of 1.7–144.5% (Fig. 9b). This frontier is the central design contribution of the BAWT framework: it transforms the binary question of “will the device work?” into a quantified, actionable trade-off surface that system designers can navigate according to the clinical priority of the monitoring task.

To appreciate the practical magnitude of these gains, consider a standard 48-hour ambulatory monitoring prescription^1,12^. Under fixed full-power operation (+ 4 dBm), the 500 mAh Li-ion cell is exhausted in 37.9 h — insufficient to complete the prescribed monitoring window. At the Pareto-optimal point of + 3.5 dBm (1.7% lifetime extension, mean Se = 93.2%), the framework already exceeds the 48-hour target. At the maximum-extension operating point (− 14.0 dBm, 144.5% lifetime extension), total operating time reaches approximately 92.6 h — more than double the monitoring window — without any hardware modification. These figures are not achievable by conventional duty-cycling strategies, which yield only 10–35% improvements while incurring latency penalties incompatible with real-time arrhythmia alerting^14,15^.

The 20% SoC operating point (Tx = − 8 dBm, BER = 1.78 × 10⁻²) is designated as the clinically critical reference throughout this work because it represents the standard firmware-defined low-battery warning threshold in commercial BLE ECG patches. At this point, BAFE achieves mean QRS sensitivity of 91.9% on MIT-BIH — with 8 of 10 individual records satisfying the ANSI/AAMI EC57 Se ≥ 95% threshold^11^ — compared to a catastrophic baseline of 35.1%. The + 56.8%-point improvement is statistically significant (McNemar test, *p*< 0.001) and represents the difference between a clinically usable device and a device that misses more than 6 in 10 QRS complexes. The 2026 systematic review by Su et al^17^. confirms that no existing WBAN power control scheme simultaneously co-designs SoC-dependent link degradation with downstream clinical signal quality, establishing that this operating-point characterization constitutes a genuinely novel contribution to the field.

The sub-Pareto point at − 14.5 dBm, which yields 154.5% lifetime extension at 43.1% mean sensitivity, is excluded from the Pareto-optimal set because a Pareto-superior point exists at − 14.0 dBm that achieves the same or higher sensitivity (45.3%) at effectively identical battery consumption. This is a consequence of the non-monotone sensitivity–BER relationship introduced by the BAFE prior: at BER values just above 6.1 × 10⁻², the morphological bandpass prior is no longer able to suppress the near-random bit-flip pattern, causing sensitivity to collapse faster than battery lifetime increases. The Pareto boundary therefore provides a principled lower bound on the minimum Tx power at which BAFE-assisted operation remains preferable to deactivating the device entirely.

### Interpretation of the BAFE mechanism and the approximate-computing trade-off

The two-stage BAFE architecture encodes a fundamental engineering insight: under severe channel degradation, the optimal receiver is not one that attempts to reconstruct the full waveform fidelity, but one that sacrifices wideband signal quality in exchange for preserving the specific morphological features — the R-peak amplitude and temporal location — that drive the clinical decision. This is the defining property of the approximate computing paradigm^33^, and BAFE is the first realization of this paradigm for BLE-degraded ECG telemetry.

The adaptive Wiener weight H_W provides a quantitative, SNR-proportional interpolation between two extreme behaviors. At high SoC (Full Power mode, γ = 23.2 dB), H_W ≈ 1 and the estimator passes the received signal nearly unmodified through the bandpass filter, providing the clean-channel diagnostic quality required for ST-segment and P-wave analysis. At critical SoC (γ = −0.8 dB), H_W = 0.4541 ≈ 0.5 and equal weight is assigned to the morphological prior and the raw observation: the prior prevents over-fitting to pervasive bit-flip noise while the raw observation preserves R-peak timing information. The resulting SQNR gain of + 3.2 dB at 5% SoC (BAFE: −2.2 dB vs. baseline: −5.4 dB) partially restores signal fidelity at the most extreme operating point. This SNR-proportional adaptation is consistent with the behavior demonstrated by Hesar and Danandeh^27^, whose adaptive Kalman filter/smoother transitions between stationary and non-stationary noise models in a structurally analogous manner.

The stage-wise SQNR decomposition at 5% SoC confirms that Stage 1 (4th-order Butterworth BPF, 0.5–40 Hz) contributes + 2.4 dB of the total SQNR gain, while Stage 2 (MMSE-Wiener weighting) adds a further + 0.2 dB. The dominant noise-rejection mechanism is therefore the morphological bandpass prior, which eliminates out-of-band bit-flip energy concentrated above 40 Hz and below 0.5 Hz. The adaptive Wiener weight provides SNR-proportional fine-tuning: at 20% SoC, removing Stage 2 while retaining Stage 1 reduces mean MIT-BIH sensitivity by approximately 3–4% points, confirming the complementary but non-dominant contribution of the MMSE-Wiener component. This two-stage decomposition validates the design choice of using a computationally lightweight closed-form Wiener weight rather than a full Bayesian inference engine: the lightweight implementation requires only 18 multiply-accumulate operations per sample and adds less than 0.08 mW of overhead^32^, whereas the DeeBayes deep Bayesian restoration network^31^ — which achieves superior noise estimation through full variational inference — imposes a computational burden that is prohibitive for the nRF52840 platform.

An important and counter-intuitive result is that BAFE increases RMSE at 20% SoC (0.250 mV BAFE vs. 0.109 mV baseline) while simultaneously improving QRS sensitivity by + 56.8 pp. This apparent contradiction is fully explained by the approximate-computing trade-off^33^: the 4th-order BPF reshapes the signal to maximize R-peak prominence within the 0.5–40 Hz band, which attenuates out-of-band energy that is part of the clean reference waveform (including high-frequency QRS slurs and low-frequency baseline components). The resulting wideband RMSE increase is a deliberate and predicted consequence of the design philosophy, not an artifact or estimation error. At 5% SoC — where bit-flip noise dominates completely — BAFE reduces RMSE from the corrupted baseline (Stage 1 alone already suppresses the dominant out-of-band noise), confirming that the RMSE trade-off is only active in the intermediate-BER regime (20–40% SoC) where channel noise and morphological distortion are of comparable magnitude. This SoC-dependent RMSE inversion is clearly visible in Fig. 5 and provides an important caution for system integrators: wideband signal quality metrics such as RMSE and PRD are not appropriate performance measures for BAFE-processed signals; per-record QRS sensitivity and the ANSI/AAMI EC57 threshold^11^ are the correct clinical endpoints.

BAFE Performance on Challenging Pathologies. Table 6 reveals that BAFE produces a net sensitivity reduction in two PTB-XL records: PTBXL-004 (Atrial Fibrillation, − 23.0 pp) and PTBXL-005 (Complete Heart Block, − 57.1 pp). Three further records show positive BAFE gains yet remain clinically sub-threshold: PTBXL-008 (Wolff–Parkinson–White, + 14.3 pp; Se = 28.6%), PTBXL-011 (Sinus Bradycardia, + 25.0 pp; Se = 75%), and PTBXL-013 (NSTEMI, + 50.0 pp; Se = 75%). In all five cases, the pathology introduces QRS morphology that departs substantially from the stationary Gaussian morphological prior assumed by the Wiener filter: irregular R–R intervals in Atrial Fibrillation cause the bandpass prior to attenuate irregularly-timed R-peaks^21^; markedly prolonged PR intervals in Complete Heart Block shift QRS energy toward the lower edge of the 0.5 Hz filter cut-off; and delta waves in WPW produce broadened, slurred QRS complexes whose energy extends beyond the 40 Hz upper boundary of the morphological band. In all five cases, the morphological prior constitutes a model mismatch that suppresses rather than enhances clinically relevant QRS components.

**Table 6:** Per-Record QRS Detection Performance—PTB-XL Dataset (SoC = 20%, BER = 1.78 × 10⁻²).

Record	Condition	SoC	BER	Sens. Base [%]	Sens. BAFE [%]	Spec. BAFE [%]	F1 BAFE [%]	ΔSens. [pp]	SQNR Base [dB]	SQNR BAFE [dB]	Clin. OK
PTBXL-001	Normal ECG	20%	1.78 × 10⁻²	8.3	100.0	100.0	100.0	+ 91.7	21.4	11.4	Yes
PTBXL-002	STEMI	20%	1.78 × 10⁻²	14.3	100.0	99.9	100.0	+ 85.7	18.7	11.7	Yes
PTBXL-003	LV Hypertrophy	20%	1.78 × 10⁻²	27.3	100.0	99.8	99.9	+ 72.7	19.6	12.8	Yes
PTBXL-004	Atrial Fibrillation	20%	1.78 × 10⁻²	69.2	46.2	60.8	52.5	−23.0	19.5	11.8	No
PTBXL-005	Complete Heart Block	20%	1.78 × 10⁻²	71.4	14.3	23.9	17.9	−57.1	18.9	12.6	No
PTBXL-006	Normal ECG	20%	1.78 × 10⁻²	25.0	100.0	99.8	99.9	+ 75.0	20.7	12.4	Yes
PTBXL-007	RBBB	20%	1.78 × 10⁻²	50.0	100.0	100.0	100.0	+ 50.0	20.4	12.0	Yes
PTBXL-008	WPW Syndrome	20%	1.78 × 10⁻²	14.3	28.6	36.7	32.1	+ 14.3	18.8	11.8	No
PTBXL-009	SVT	20%	1.78 × 10⁻²	40.0	100.0	100.0	100.0	+ 60.0	18.8	11.9	Yes
PTBXL-010	Normal ECG	20%	1.78 × 10⁻²	7.7	100.0	99.9	100.0	+ 92.3	18.5	11.3	Yes
PTBXL-011	Sinus Bradycardia	20%	1.78 × 10⁻²	44.4	77.8	92.6	84.6	+ 33.4	19.6	13.1	No
PTBXL-012	Atrial Flutter	20%	1.78 × 10⁻²	23.1	100.0	99.7	99.8	+ 76.9	18.7	12.3	Yes
PTBXL-013	NSTEMI	20%	1.78 × 10⁻²	25.0	75.0	100.0	78.7	+ 50.0	19.7	11.5	No
PTBXL-014	Premature Atrial Complexes	20%	1.78 × 10⁻²	27.3	100.0	100.0	100.0	+ 72.7	20.4	11.5	Yes
PTBXL-015	Normal ECG	20%	1.78 × 10⁻²	46.2	100.0	100.0	100.0	+ 53.8	18.9	11.4	Yes
Mean (20% battery)	—	20%	1.78 × 10⁻²	32.9	82.8	86.5	84.4	+ 49.9	19.5	12.0	10/15

Notation and abbreviations as in Table 3. Per-record sensitivity values reflect 7–25 beats per record, inherent to the PTB-XL 10-second acquisition protocol; see the section “Simulation protocol”.

These five records collectively define the operational boundary of BAFE: the estimator is beneficial and clinically reliable for stationary, regular-morphology conditions (Normal Sinus Rhythm, LBBB, SVT, Atrial Flutter, Sinus Tachycardia, Sinus Bradycardia with regular morphology, and most STEMI/NSTEMI presentations with preserved R-peak prominence) and harmful for highly irregular or morphologically atypical conditions (AF, CHB, WPW, and any condition where QRS energy is concentrated outside the 0.5–40 Hz band). This boundary is consistent with the QRS morphological classification reported by Prakash et al^20^., who identify irregular-morphology arrhythmias as the primary failure mode of fixed-prior ECG estimators, and aligns with the clinical finding of Lim et al^21^. that Atrial Fibrillation requires beat-wise adaptation of the detection algorithm rather than a stationary filter bank. The most direct remediation — a pathology-aware prior that selects the appropriate morphological template from a pre-trained arrhythmia library — is identified as a planned extension in the section “Limitations and future directions”. Until such an extension is validated, clinical deployment of BAFE should incorporate a rhythm-irregularity classifier: if the inter-beat interval coefficient of variation exceeds a pre-defined threshold (e.g., > 0.15 for AF detection^21^), the controller defaults to passing the unfiltered received signal, bypassing the Wiener prior entirely.

Regarding the two sub-threshold MIT-BIH records (MITBIH-005, PVC; MITBIH-009, VT): both conditions involve non-stationary QRS morphology that challenges the Pan–Tompkins detector^10^ even on clean, uncorrupted signals. At 20% SoC, BAFE improves sensitivity by + 41.1 pp (PVC: 31.2% → 72.3%) and + 31.7 pp (VT: 37.4% → 69.1%), confirming that BAFE substantially mitigates the BLE-induced degradation even for these challenging morphologies. The sub-threshold result reflects an inherent limitation of the Pan–Tompkins detector under polymorphic QRS waveforms rather than a failure of the BAFE recovery stage: substituting an adaptive QRS detector — such as the CNN-based architectures benchmarked in^19^ — is expected to close this gap.

### Contextual comparison with prior work

Table 5 provides a structured benchmark of the BAWT framework against five representative prior methods. The comparison is organized around each method’s actual documented contributions, since no prior method jointly evaluates BLE Tx power reduction, battery SoC modeling, and clinical QRS detection accuracy under the same framework — confirming that the co-design problem addressed by BAWT remains unresolved in the literature, as corroborated by three independent 2026 analyses^17,23,24^.

The 2024 adaptive BLE Tx power control scheme of Salomon and Boano^15^ is the most closely related work on the transmission side: it demonstrates that per-channel Tx adjustment based on RSSI feedback achieves up to 50% energy saving and substantially improves packet reception ratio — a finding consistent with the adaptive power control component of BAWT. However^15^, does not model SoC-dependent power reduction, does not characterize the resulting BER elevation under Rayleigh fading, and reports no clinical ECG quality metric. The BAWT framework extends this contribution by explicitly linking the SoC-to-Tx-power mapping (via Peukert-corrected coulomb integration) to the BER at the receiver, and by demonstrating that receiver-side processing — not just Tx-side adaptation — is necessary to maintain clinical signal quality when the link is already degraded.

Duty-cycle scheduling^6,14^ achieves 10.53–35% energy reduction through sleep-mode activation, but at the cost of data gaps that are incompatible with continuous arrhythmia monitoring. Neither study evaluates QRS sensitivity under degraded channel conditions or models the SoC-to-BER coupling that is the root cause of the failure mode addressed here. ECG compression via approximate message passing^16^ achieves superior PRD and RMSE values but operates on a clean-channel assumption and reports no QRS detection metric. Deep learning-based ECG denoising^19^ achieves 97.31% accuracy for noise-type classification (baseline wander, electrode motion, muscle artifacts) but assumes clean packet delivery; it neither models BLE bit-flip corruption nor addresses battery-aware power control. Critically, as confirmed by the 2026 arrhythmia detection study of Sundari et al.^23^, current embedded deep learning frameworks for wearable ECG explicitly exclude the BLE link degradation scenario from their evaluation protocols, further establishing the novelty boundary of the present work.

Quantitatively, BAFE achieves a 2.6× higher mean QRS sensitivity than the best-performing competing method at the same 20% SoC operating point (91.9% vs. 35.1% unassisted baseline; no prior method reports a comparable metric under degraded BLE link conditions). The 144.5% maximum battery lifetime extension achieved by the adaptive power controller exceeds the 10–50% range reported by duty-cycling and per-channel BLE adaptation strategies^6,15^, while maintaining clinically acceptable QRS detection for the majority of arrhythmia types. BAFE is therefore the only approach in the comparative analysis to simultaneously provide: (i) six-state SoC-aware Tx control with Peukert-corrected discharge modeling; (ii) Rayleigh-BER-tolerant receiver processing validated on two independent clinical databases under the ANSI/AAMI EC57 standard; (iii) per-record QRS sensitivity reporting at each of seven SoC levels; and (iv) a fully characterized Pareto-optimal design envelope linking BER tolerance, sensitivity, and battery lifetime extension.

### Limitations and future directions

Acknowledged Limitations. The following limitations bound the scope of the present validation and should be addressed in future extensions:

- *Channel Model Scope*: The patient–receiver distance is fixed at d = 3 m with a static body-tissue attenuation of 15 dB and an indoor multipath margin of 20 dB. Mobility-induced Doppler spread, dynamic body shadowing, and furniture-scattered multipath — all of which alter the instantaneous Rayleigh fading statistics — are not modeled. The BER values reported in Table 2 are therefore worst-case analytical bounds rather than time-averaged field measurements. Real-world deployment will require an empirical channel characterization campaign consistent with the methodology of^8^ and the BLE body-coupling analysis of^4^.
- *Stationarity Assumption*: The BAFE morphological prior assumes stationarity of the ECG signal within each processed frame. This assumption is violated during sustained ventricular arrhythmias (PVC, VT), highly irregular atrial rhythms (AF), and conduction defects with markedly prolonged PR intervals (CHB), as evidenced by the sub-threshold MIT-BIH records (MITBIH-005, MITBIH-009) and the BAFE-degraded PTB-XL records (PTBXL-004, PTBXL-005, PTBXL-008). For these conditions, the fixed 0.5–40 Hz prior constitutes a model mismatch that should be replaced by a pathology-adaptive prior^19,28^.
- *Beat-Count and EC57 Compliance*: Full compliance with the ≥ 200-beat evaluation protocol of ANSI/AAMI EC57^11^ is satisfied for all 10 MIT-BIH records (1,714–2,384 annotated beats per 30-minute recording). PTB-XL records are inherently limited to 7–25 beats per 10-second diagnostic snapshot — a constraint of the PTB-XL acquisition protocol, not the simulation design. Per-record PTB-XL sensitivity estimates therefore have a discrete resolution of 1/N and should be interpreted as proof-of-concept BER-sensitivity indicators; the primary statistical evidence derives from the aggregate of 15 records × 7 SoC levels = 105 evaluated conditions and is further supported by the McNemar test (*p* < 0.001).
- *BLE Protocol Modeling*: The simulation evaluates Rayleigh bit-flip corruption as an independent, memoryless process applied uniformly to all transmitted bits. In practice, BLE 5.0 employs FHSS (Frequency Hopping Spread Spectrum) across 37 data channels, 24-bit CRC per packet, and an ARQ retransmission scheme^38^. The bit-level corruption model adopted here represents a worst-case analytical bound applicable when retransmission opportunities are exhausted — for example, under sustained link degradation, buffer overflow, or hard latency deadlines^39^. The gap between this worst-case bound and typical operation under the BLE ARQ scheme remains to be characterized experimentally. A dedicated future extension will implement a packet-level simulation of the BLE MAC Layer 2 (including CRC checking, ARQ retransmission scheduling, and buffer-overflow modelling), enabling a rigorous quantitative comparison between the worst-case bit-level bound adopted here and the realistic per-packet delivery behaviour of a compliant BLE 5.0 stack.
- *Hardware Validation*: The BAFE evaluation is conducted exclusively in software simulation. On-device power consumption, interrupt latency, and memory footprint of the nRF52840 implementation have not been measured. The theoretical estimate of < 0.08 mW overhead requires empirical verification on physical hardware.

Planned Extensions. The following four extensions will address the limitations above and expand the framework toward clinical deployment:

- *Extension 1—In Vivo Validation*: In vivo clinical validation with a hardware BLE ECG patch on consented volunteers across controlled indoor environments, following the preclinical wearable validation protocol of Ahmmed et al.^43^. This phase will also provide the first empirical channel characterization data under realistic body-coupled BLE operation^4^, supplanting the analytical worst-case channel model and enabling full ANSI/AAMI EC57 compliance evaluation on PTB-XL-class 10-second segments via multi-record aggregation across extended monitoring sessions.
- *Extension 2—Pathology-Adaptive Prior*: Replacement of the fixed Wiener prior with a pathology-adaptive deep learning morphological prior^19,28^ that modulates the filter bandwidth and morphological template in real time according to an on-device rhythm classification output. This extension directly addresses the five sub-threshold PTB-XL pathologies identified in the section “Interpretation of the BAFE mechanism and the approximate-computing trade-off” and is motivated by the 2026 demonstration^31^ that interpretable Bayesian priors combined with learned feature extraction consistently outperform fixed-prior estimators under severe signal corruption.
- *Extension 3—Multi-Hop and Energy Harvesting Integration*: Extension to body-area-network multi-hop relay topologies^5,15,44^ and integration with energy-harvesting wearable architectures^45^. The 2026 review by Su et al^17^. identifies energy harvesting and cooperative relay as the two highest-impact mechanisms for further extending WBAN operational lifetime beyond the limits of battery-based systems; integrating BAWT with these mechanisms would enable perpetual or near-perpetual monitoring without recharging. The AI-driven health-aware control framework of Jain and Bhullar^18^ provides a complementary system-level architecture for integrating power management, link quality control, and clinical decision support within a unified wearable platform.
- *Extension 4—TinyML Hardware Implementation*: Hardware implementation of the BAFE pipeline on a TinyML processor using approximate arithmetic^29,33^. The nRF52840 Cortex-M4F core executes the 4th-order IIR filter and Wiener weighting in under 2 µs per sample at 64 MHz^32^; a dedicated TinyML accelerator with approximate multipliers^29^ is expected to reduce this to sub-microsecond execution while cutting arithmetic energy by a further 40%. This extension will also characterize the robustness of the quantized BAFE implementation to fixed-point rounding errors across the six SoC operating modes.

## Conclusion

Continuous cardiac monitoring via wearable BLE-enabled ECG patches is undermined by a critical and clinically dangerous failure mode: as battery charge depletes, mandatory reductions in RF transmission power corrupt the received signal to a degree that renders conventional QRS detection unreliable at precisely the moment monitoring is most vital.

This paper has addressed this vulnerability through the Battery-Aware Approximate Wireless Telemetry (BAWT) framework, which co-designs a Li-ion discharge model, a six-state adaptive RF power controller, and a physically motivated 2.4 GHz ISM band indoor channel model. At the receiver, the Bayesian Adaptive Feature Estimator (BAFE) recovers clinically meaningful QRS features from severely corrupted bit streams without any forward error correction hardware.

Validation on the MIT-BIH Arrhythmia and PTB-XL databases confirms the framework’s efficacy. At 20% SoC — the most clinically critical operating condition — BAFE achieves mean QRS sensitivity of 91.9% and 82.8% on the two databases respectively, representing improvements of 56.8 and 49.9% points over the unassisted baseline (35.1% and 32.9%, respectively). At the individual-record level, 8 of 10 MIT-BIH and 10 of 15 PTB-XL records satisfy the ANSI/AAMI EC57 clinical acceptability threshold, confirming viability across diverse arrhythmia morphologies. Beyond signal recovery, the adaptive power controller extends the critical operating window by 348 min (355%) over fixed full-power operation, while the fully characterized Pareto-optimal region (spanning − 14.0 to + 3.5 dBm) achieves mean QRS sensitivity of 45.3–93.2% across the trade-off frontier, with battery lifetime extensions of up to 144.5%; at the most critical 20% SoC, 8 of 10 MIT-BIH records individually satisfy the ANSI/AAMI EC57 clinical threshold.

Collectively, these results demonstrate that robust cardiac monitoring under severe power constraints is achievable through principled co-design of battery management, RF control, and approximate signal processing — without hardware modifications. Future work will pursue in vivo validation on ambulatory subjects, extension to multi-lead patch architectures, and integration of on-device arrhythmia classification to further bridge the gap between laboratory validation and clinical deployment.

## Data Availability

This study utilized two publicly available electrocardiography databases. The primary training and evaluation dataset is the PTB-XL electrocardiography database (PhysioNet). Dataset access: https://physionet.org/content/ptb-xl/1.0.3/— DOI: https://doi.org/10.13026/kfzx-aw45. The external validation dataset is the MIT-BIH Arrhythmia Database. Dataset access: https://physionet.org/content/mitdb/1.0.0/— DOI: https://doi.org/10.13026/C2F305. Both datasets are openly accessible and were used in accordance with their respective terms of use.
